# Management of Nephrotic Syndrome in Pediatric Patients Treated by Different Steroid Regimens

**DOI:** 10.3390/medicina61071257

**Published:** 2025-07-11

**Authors:** Valeria Chirico, Filippo Tripodi, Giovanni Conti, Lorena Silipigni, Antonio Lacquaniti, Paolo Monardo, Roberto Chimenz

**Affiliations:** 1 Pediatric Nephrology and Dialysis Unit, University Hospital “G. Martino”, 98124 Messina, Italy; 2 Nephrology and Dialysis Unit, Papardo Hospital, 98124 Messina, Italy

**Keywords:** idiopathic nephrotic syndrome, steroid-sensitive nephrotic syndrome, minimal change disease

## Abstract

*Background and Objectives*: The nephrotic syndrome (NS) is the most common acquired childhood kidney disease. Steroids represent the cornerstone of the therapeutic strategy, representing the first-line approach, but optimal therapeutic management is debated. This study aimed to compare different steroid therapeutic management protocols. *Patients and Methods*: A total of 140 NS pediatric patients were enrolled retrospectively. All the kids were divided among three different groups according to the three different steroid therapeutic schemes: 2240 mg/m^2^ (group 1), 3360 mg/m^2^ (group 2), or 3640 mg/m^2^ (group 3) and divided in frequently relapsing (FR-NS) or steroid-dependent (SD) NS. *Results*: Within group 1, 50% of the population developed FR-NS; 100% of those kids were between 2 and 6 years old. Within the second group, 54% of the patients developed FR-NS, and 83% of these kids were between 2 and 6 years old, i.e., 45% of the group population. Within group 3, 45% of the patients developed FR-NS, and 70% of these kids were among 2 and 6 years old, i.e., 32% of the group population. This group exhibits the lowest percentage (42%) of patients in the highest relapse category (≥5 relapses) compared to the other protocols, indicating that this protocol might be more effective at reducing the number of frequent relapses. No specific predictor factors of FR- or SD-NS were revealed in the studied cohort. *Conclusions*: A longer steroid scheme does not correlate with a better outcome, nor does it reduce the number of relapses or prevent steroid failure.

## 1. Introduction

Idiopathic nephrotic syndrome (NS) is the most common acquired childhood kidney disease and is one of the most common glomerular disorders in childhood, with an incidence of 2–7 cases per 100,000 children and a prevalence of nearly 16 cases per 100,000 [1,2].

NS consists of an ensemble of signs and symptoms, with the leading ones consisting of periorbital, perineal, and ankle swellings related to a higher proteinuria rate [3,4].

Commonly, podocyte diaphragm damage causes protein loss with urine outflow, leading to a change in oncotic pressure. This results in soft tissue edema and consequently swallowing [5].

The main cause of NS in children is represented by minimal change disease, characterized by minimal alterations in the glomerulus observed by light microscopy with little or no Ig deposition. The major histologic feature of minimal change disease remains the foot process effacement of the podocytes that can be highlighted by electron microscopy.

Less commonly, a biopsy-based diagnosis of focal segmental glomerular sclerosis in patients with idiopathic nephrotic syndrome has been made.

Steroid-sensitive (Ss) NS underlying minimal change disease usually tends to respond well to steroid treatment and is a positive prognosis hallmark.

However, some patients with minimal change disease will not respond well to the steroid treatment and will develop a steroid-resistant (Sr) NS, and conversely, a minority of patients having focal segmental glomerular sclerosis lesions respond to steroids.

Indeed, many authors states that podocyte injury is a board-spectrum disorder starting with a reversible minimal disruption of the podocyte foot cytoskeleton leading to the increased permeability of the slit diaphragms and consequently albuminuria, ending eventually with podocyte apoptosis and the resulting glomerulosclerosis hallmark of focal segmental glomerular sclerosis [6].

Even though the morphologic features and pathology mechanisms have been well analyzed in the scientific literature, further studies should highlight the possible etiopathogenic mechanisms underlying podocyte injury.

Podocyte diaphragm alteration is the main cause of the clinical features of NS, leading to nephrotic-range proteinuria and albuminuria. As a result of protein loss in urine flow, NS onsets with periorbital or scrotal edema or that of the lower limbs.

Two different mechanisms have been identified as being responsible for contributing to nephrotic edema: the underfilling void mechanism, as previously cited, and the overfilling mechanism due to resistance to atrial natriuretic peptide (ANP) and the activation of epithelial sodium channels (ENaCs), leading to an expansion of the intravascular compartment and consequently edema [7].

The definition of Ss-NS has been modified by the International Pediatric Nephrology Association (IPNA) through expert members’ consensus over the years. As an appropriate definition, Ss-NS is now better defined as an idiopathic disease responsive to 4 weeks of corticosteroid therapy with or without a confirmation period of 2 additional weeks [8,9].

Although Ss-NS is a disease with a wide range of options in treatment management, steroids represent the cornerstone of its therapeutic strategy, having been used as the first-line approach over the past decades till today [10,11].

Steroid use and efficacy are not a matter of debate, but the optimal therapeutic management of NS at onset has been questioned for a long time, being attributed to the clinical experience of each center [12].

The Italian Society of Pediatric Nephrology (SINePe) guidelines have outlined the pathway used for different steroid therapeutic schemes used over the years [13].

The first protocol adopted consisted of an eight-week steroid scheme (4 + 4) suggested by the International Study of Kidney Disease in Children (ISKDC) that agreed on an empirical dose of steroids of 60 mg/m^2^/day (maximum dosage: 80 mg/24 h) in divided doses for four weeks, followed by 40 mg/m^2^/day in divided doses for three consecutive days out of seven, as the standard treatment for the first episode of NS. The established regimen was proposed based on ISKDC members’ clinical experience and the reviewed literature; it consisted of a daily prednisone or prednisolone dose for four weeks followed by corticosteroids given on three consecutive days out of seven for four weeks [14].

Over time, the first Cochrane meta-analysis was published, and in 2000, a 3-month steroid therapy minimum was suggested, resulting in fewer children relapsing by the 12–24-month follow-up [15].

This recommendation was further defended by the KDIGO 2012 guidelines, suggesting a daily oral prednisone of 60 mg/m^2^ given for 4–6 weeks, followed by alternate-day medication for 2–5 months with tapering of the dose to 40 mg/m^2^ (or 1.5 mg/kg) [16].

The KDIGO 2012 guidelines were widely adopted as the standard management approach for Ss-NS over recent years [17].

This was the state of the art until Teeninga revealed that long-term steroid treatments at onset did not improve clinical outcome [18]. More recently, Cochrane systematic reviews challenged the previous treatment as no significant difference was found in 6 months of prednisolone treatment compared to 3 months of prednisolone treatment [19,20].

Prednisone dosage using weight or body surface has been used interchangeably over time, and a prednisone dose of 60 mg/m^2^ is considered equivalent to 2 mg/kg, although this approximation underestimates the dose per body surface area in smaller children [21,22].

A simplified formula for a dose of 2 mg/kg + 10 has been shown to be an accurate approximation of the prednisone dose, calculated in mg/mg^2^, if not exceeding 60 mg/day [23].

In Italy, a multi-center, retrospective study conducted by Pasini showed differences in NS steroid management at onset, since shared guidelines were missing when different therapeutic approaches started being used [24].

Based on the mentioned experiences and the scientific literature, we conducted a retrospective single-center study evaluating the treatment and management of Ss-NS, comparing three different steroids’ therapeutic management protocols carried out over three decades. The identification of risk factors for relapsing and steroid-dependent nephrotic syndrome was performed.

## 2. Materials and Methods

A total of 140 pediatric NS patients followed up at the Pediatric Nephrology and Dialysis Unit of the “G. Martino” University Hospital of Messina were enrolled retrospectively between 1 January 1994 and 31 May 2024.

Every patient had to undergo at least a 24-month follow-up, had received an NS diagnosis, and had a complete medical record.

Diagnostic criteria for nephrotic-range proteinuria were defined based on protein present in urine: a creatinine ratio (UPCR) of at least 200 mg/mmol (2 mg/mg) in the first morning urine void or proteinuria of at least 40 mg/m^2^/h or 50 mg/kg/die (1000 mg/m^2^ per day) in a 24 h urine collection corresponding to a +++ (300–1000 mg/dL) or ++++ (>1000 mg/dL) in a urine dipstick. NS was defined as the presence of nephrotic-range proteinuria associated with a serum albumin concentration lower than 30 g/L, with or without edema, or edema when albumin was not evaluated.

Patients with an Ss-NS diagnosis were grouped together according to the following IPNA definition: NS responsive to 4 weeks of corticosteroid therapy with or without a confirmation period of 2 additional weeks. All patients were treated with the first-choice steroid option, based on a single morning oral dose of prednisone [2].

No other steroids, such as deflazacort, dexamethasone, betamethasone, and methylprednisolone, were used in treatments.

The right steroid daily dosage was calculated using the body surface area using the Mosteller formula, as the most recent guidelines state. The most recent formula was used to estimate a dose of 60 mg/m^2^ [2 × W + 8] and a dose of 40 mg/m^2^ was [W + 11] [13].

Exclusion criteria consisted of a diagnosis of secondary, steroid-resistant, drug-related, or chronic kidney disease (CKD)-related NS. Patients with partial medical record information were excluded. Moreover, patients who did not complete the steroid protocol were not included in the statistical analyses and excluded from this study. A total of 8 patients received an Sr-NS syndrome diagnosis, 1 patient presented with iatrogenic NS, and 1 patient with CKD-related NS. A total of 10 patients had a follow-up of less than 24 months. All previous patients were excluded, with 120 pediatric patients enrolled for statistical analyses.

All these kids were divided according to three different steroid therapeutic schemes:

A total of 40 patients (8 weeks—8 W) were treated according to an eight-week regimen scheme as stated in the ISKDC protocol, with an average total cumulative steroid dose equal to 2240 mg/m^2^ [14]. The other 40 patients received a twelve-week regime scheme, as stated in the SINePe guidelines, with an average total cumulative steroid dose equal to 3360 mg/m^2^ (12 weeks—12 W) [13]. The remaining 40 patients were treated for 4 months according to KDIGO 2012 protocol guidelines, with a total of 20 weeks of steroid regimen including the decalage and an average total cumulative steroid dose equal to 3640 mg/m^2^ (20 weeks—20 W) [17].

### 2.1. Outcomes

To compare the three different protocols adopted over the years according to the current guidelines, relapsing nephrotic syndrome (FR-NS) and the steroid-dependent nephrotic syndrome (SD-NS) were evaluated. In particular, FR-NS occurred when two or more relapses were detected in the first 6 months following the remission of the first episode, or three or more relapses in a period of 12 months; conversely, SD-NS is characterized by two or more consecutive relapses during the recommended steroid therapy on onset or relapse or within 14 days of its discontinuation [10].

Relapses were defined as 3+ or 4+ on a urine protein dipstick or using the UPr/UCr ratio. A UPr/UCr ratio of > 200 mg/g (20 mg/mmol; 2 g/g) in two 24 h urine samples was the major criterion for defining relapses [25].

The established outcomes were defined as the most useful to evaluate the steroid response since they have been reported in literature data and have been validated as well-representative outcomes to steroid protocol efficacy [26].

Other clinical features were finally analyzed in our population such as sex, onset of side effects during steroid treatment, and fever onset during the first relapse to provide further data about FR-NS and SD-NS patients.

### 2.2. Statistical Analysis

Statistical analyses were performed with the SPSS 24 (SPSS Inc., Chicago, IL, USA) software and the GraphPad Prism (version 9.4.1; GraphPad Software, Inc., San Diego, CA, USA) package. Data were presented as mean ± SD for normally distributed values (from the Kolmogorov–Smirnov test) and median IQ range for non-normally distributed values.

Differences between groups were established by an unpaired Welch *t*-test, chi-squared test, or ANOVA followed by Bonferroni’s test for normally distributed values and by Kruskal–Wallis’s analysis followed by Dunn’s test for nonparametric values.

Survival data were analyzed using the Kaplan–Meier method and compared using the log rank test.

## 3. Results

Table 1 presents the general features of the population cohort. Patient characteristics at disease onset were similar in the three cohorts.

Our cohort was composed of 87 males (73%) and 33 females (27%).

In the male population, 70 patients had a first onset of NS between 2 and 6 years old.

A total of 44 male patients developed SD- or FR-NS, and 51% of them had a relapse between 2 and 6 years of age. Overall, 18% of male patients experienced side effects during the first steroid treatment. Conversely, 15 female patients developed SD- or FR-NS. A total of 24 (73%) children had their first onset of NS between 2 and 6 years of age. Furthermore, 38% of female patients experienced side effects during the first treatment.

Overall, 94 patients (78%) were between 2 and 6 years of age, and 26 patients were older (22%). A total of 59 patients (49%) had an FR-NS diagnosis. Moreover, 25 patients (21%) experienced side effects during steroid treatments, 95 patients did not experience any side effects (79%).

In SD-NS patients, other immunosuppressive drugs were administered, alone or in combination, such as cyclophosphamide, rituximab, cyclosporine, tacrolimus, and mofetil mycophenolate.

### 3.1. Relapse Incidence Among Protocols

Overall, 50% of patients treated according to the ISKDC protocol (8 W) developed FR-NS.

The mean number of relapses was 3.2 ± 2.9 during the follow-up period, with an average number of asymptomatic days after onset equal to 130 days.

In the 12 W group, treated according to the SINEPE protocol, 44% of patients developed FR-NS, and 72% of them were between 2 and 6 years old. The average relapse number was equal to 3.2 ± 2.3 during the follow-up, with an average number of asymptomatic days after onset equal to 120 days. Overall, 45% of patients in the 12 W group experienced four or more relapses.

Moreover, 55% of patients treated according to the KDIGO 2012 guidelines (20 W) developed FR-NS, and 82% of them had an age between 2 and 6 years.

The average relapse number was equal to 4.2 ± 2.9 during the follow-up, with an average number of asymptomatic days after onset equal to 152 days. About 59% of the analyzed population experienced four or more relapses.

In our population, relapses were especially observed during the first ten weeks, independent of the protocol used.

**Figure 1:**
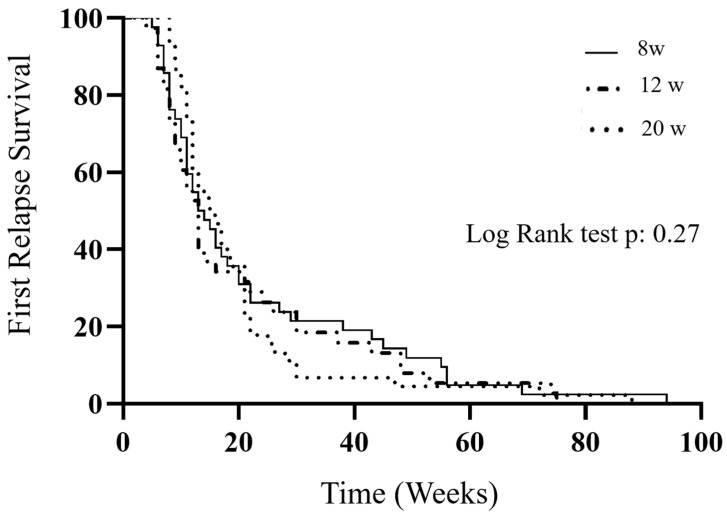
The comparison of the first relapse in the three groups according to steroid regimen.

The median time to first relapse was estimated by the Kaplan–Meier survival curve. It was 13 weeks [95% C.I.: 8.5–24.5] in the 8 W group, 16 weeks [95% C.I.: 12–21] in the 12 W group, and 13 weeks [95% C.I.: 8–27] in the 20 W group. No differences were assessed comparing the three groups according to the steroid treatment (*p*: 0.27) (Figure 1).

### 3.2. Number of Relapses and Remission

The 20 W protocol showed a high percentage (54%) of patients in the highest relapse category (≥4 relapses), suggesting low efficacy in preventing frequent relapses. However, no statistical difference was found between a longer steroid treatment and a lower number of relapses.

Conversely, the 8 W protocol was characterized by the lowest percentage (42%) of patients in the highest relapse category (≥5 relapses) compared to the other protocols, indicating that the 8 W protocol might be more effective in reducing the number of frequent relapses.

The 12 W protocol has a balanced distribution among all relapse terciles, indicating a lower response variability to treatment.

In our population, most of the relapses happened during the first ten weeks regardless of the protocol used.

No statistical significance was found when the asymptomatic days from the end of therapy to the first relapse among the three different protocols was compared.

**Figure 2:**
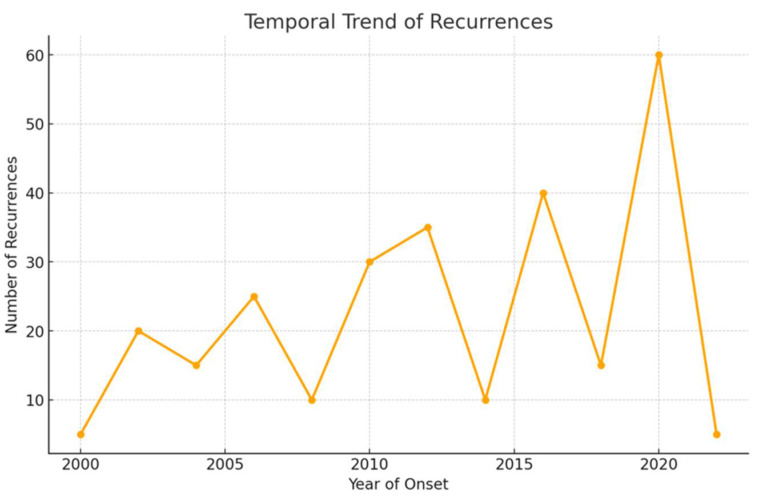
The number of relapses occurring during the follow-up period.

Figure 2 illustrates the number of relapses recorded over the years of enrollment and follow-up, according to the onset year of NS, underlying a peak of relapses during the Coronavirus-19 pandemic.

### 3.3. Risk Profile of NS

Overall, 61% of Ss-NS patients had a febrile first relapse when compared to 39% of FR-NS patients. Furthermore, 85% of FR-NS patients that were 6 years old had no fever when compared to 63% of FR-NS patients who were younger.

Furthermore, 20 out of 25 (80%) patients who experienced side effects during the first-line treatment developed FR- or SD-NS.

Among the 95 patients who did not experience side effects, 56 patients developed Ss-NS and 39 developed FR- or SD-NS. Patients experiencing side effects during the first-line treatment more frequently developed FR- or SD-NS.

## 4. Discussion

This study revealed that three different steroid strategies did not impact the relapse rate in patients developing FR- or SD-NS during a follow-up period of two years. Longer steroid regimens, such as the 12 and the 20 W protocols, did not correlate with a better outcome in terms of the relapse rate, average number of relapses, or incidence of FR- or SD-NS. Although the 8 W regimen exhibited the lowest percentage of patients with five or more relapses, if compared to other steroid treatments, this therapeutical scheme did not maintain a long remission period when compared to the other two groups, considering that 50% of patients developed FR/SD-NS, suggesting that this protocol may not be sufficient for maintaining long-term remission. However, no statistically significant differences were found when the three different steroid protocols were compared.

The best therapeutic strategy should not be based on the total amount of steroid administered or a longer steroid exposition, considering that the 20 W regimen (3640 mg/m^2^ of steroid administered) did not produce a reduction in the relapse rate or FR-/SD-NS diagnosis, when compared to the 8 W strategy (cumulative steroid dose: 2240 mg/m^2^).

Furthermore, our data confirmed that the protocol proposed by the SINePe, widely used in the Italian setting, produced, with a lower dosage of steroids, the same outcomes in terms of the relapse rate, average number of relapses, patients without relapses, and FR- or SD-NS diagnosis rate, when compared to the 20 W protocol [13,18,19].

**Figure 3:**
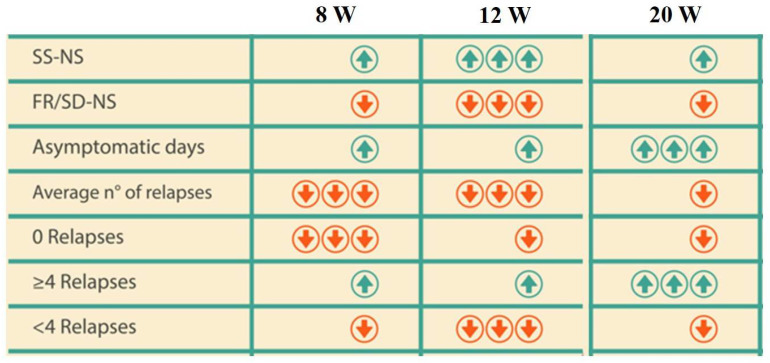
The potential effects of steroid regimens on the relapse rate. Abbreviations: W: weeks; SS-NS: steroid-sensitive nephrotic syndrome; FR/SD-NS: frequently relapsing/steroid-dependent nephrotic syndrome; 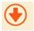 low negative probability; 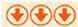 high negative probability; 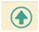 low positive probability; 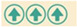 high positive probability.

By analyzing our cohort, we found that the 12 W protocol could be a better therapeutic option since it has a higher tendency to lower the risk of relapses and steroid dependence, with a better sustainability of NS over time, as a longer treatment does not produce a better steroid response or a lower relapse number (Figure 3).

This study is aligned with the current literature, as Teeniga suggested that a treatment longer than three months does not bring any other benefits in terms of reducing the relapse rate [18]. Our data underlined that just 54% of patients treated with a longer steroid course, against 45% treated with a shorter course, developed steroid failure.

Our data confirm that a longer steroid treatment does not contribute to reducing relapses, as most of the patients treated with a longer course experienced a higher number of relapses, with five or more relapses, strengthening the literature data [19,27].

Pasini stated that a good optimization of the therapeutic strategy was needed in Italy, since the standardized treatment introduced by SINePe led to better management, outcomes, and suitability of NS for pediatric patients, reducing the number of relapses and steroid-dependent or frequently relapsing patients [13]. Patients treated according to the KDIGO 2012 guidelines exhibited a higher number of relapses and a higher number of FR- and SD-NS diagnoses in the follow-up period.

These data showed that a longer treatment could maintain a longer asymptomatic period without an optimal control on relapses and without altering the natural history of the disease during a long follow-up period.

In our population, most of the relapses happened during the first ten weeks regardless of the protocol used, with a prevalence of males over females, confirming the gender tendency observed in other reports [28,29,30].

The second part of our study identifies the possible clinical risk factors for FR/SD-NS diagnosis.

In our cohort, male patients with an onset of NS between 2 and 6 years of age more frequently developed FR- or SD-NS, whereas male patients with NS onset at an age of 6 years or older more frequently developed Ss-NS. Conversely, female patients with an NS onset between 2 and 6 years of age more frequently developed Ss-NS. The literature states that male patients with an earlier disease onset are at a higher risk of developing steroid dependency and having frequent relapses [31].

However, female patients with an NS onset at or after 6 years of age are more likely to develop FR- or SD-NS. These gender differences show that genetic and biological factors play a role in nephrotic syndrome onset.

However, the early identification of FR- or SD-NS represents a significant unmet clinical need due to the absence of a predictive model to facilitate a precocious diagnosis. In our studied population, patients experiencing side effects during steroid treatment developed a significant association with FR/SD-NS. This correlation reflects an individual predisposition, influences steroid response, and needs further studies for a deeper personalization of therapeutic strategy.

Viral and upper respiratory infections, the well-documented triggers of the possible relapses predisposed by steroid therapy, alter both innate and adaptive immune responses, with lymphocytes, particularly T and B cells, being central to the occurrence of relapses [32,33].

Recently, The Kidney Disease: Improving Global Outcomes (KDIGO) Clinical Practice Guideline for the Management of Glomerular Diseases states that, for children with FR- or SD-NS, daily steroids should not be administered during the episodes of upper respiratory tract and other infections to reduce the risk of relapse, confirming the pathogenetic role of infection, inflammation, immune dysfunction, and steroid therapy [34].

This phenomenon activates the transcriptional and cytokine pathways, inducing inflammatory mechanisms closely related to NS relapses, and is highlighted by several markers, such as increased platelet counts and acute-phase reactants like C-reactive protein, interleukin-6, and tumor necrosis factor-alpha [35].

Systemic inflammation and an altered immune system are closely related to renal dysfunction and poor prognosis, revealed in clinical practice by proteinuria, a crucial marker for steroid responsiveness.

Moreover, the dysregulated lipid metabolism, induced by prolonged steroid therapy and assessed by lipidomics and proteomics analyses, was related to proteinuria severity, suggesting that lipid dysregulation, highlighted by altered adipokine or low-density lipoprotein (LDL) cholesterol levels, may play a central role in NS progression [36].

In contrast with the current literature, we found that patients experiencing a febrile episode followed by their first relapse more frequently showed a tendency to develop Ss-NS; otherwise, the first non-febrile relapse was followed by steroid failure in the long term. These data could show that genetic and immunological factors, more than others, could play a role in steroid response. This datum could be related to a “false relapse”, observed during an infectious disease, which could temporarily induce the worsening of the renal function or urine protein levels. This reversible condition could explain the association with a febrile episode of relapse, followed by a resolution of NS. Conversely, a “true relapse” is not associated with febrile episodes, inducing the irreversible worsening of NS.

**Figure 4:**
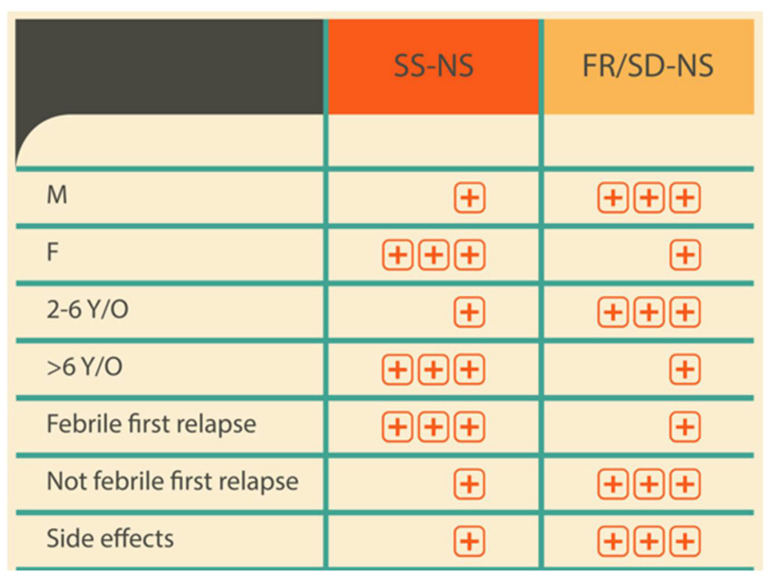
The potential risk factors of relapse and steroid response in nephrotic syndrome. Abbreviations: SS-NS: steroid-sensitive nephrotic syndrome; FR/SD-NS: frequently relapsing/steroid-dependent nephrotic syndrome; M: male; F: female; Y/O years old; 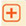 = low risk; 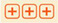 = high risk.

Figure 4 presents the epidemiological data observed in our population during the follow-up period, highlighting the potential risk related to each factor, such as gender, age, or side effects related to steroid therapy.

This study has several limitations. The retrospective analysis and the relatively small number of enrolled patients represent the main study limitations. Moreover, the data about associations between sex, age at onset, side effects, fever, and disease progression should be confirmed in a wider cohort. 

This study described the epidemiological impact of these parameters in the enrolled population, receiving the NS diagnosis between two and six years of age, a common age of idiopathic nephrotic syndrome onset.

However, highlighting a significant overlap between our cohort study characteristics and the literature data, this studied cohort could reflect a well-representative general population, but our reports described the epidemiological data, highlighting that multivariate adjustments and regression analyses were not performed to statistically establish risk factors and rule out confounding bias.

During the follow-up period, the absence of genetic and molecular markers did not allow for assessing the risk factors that could influence the therapy response. Larger studies are needed to explore potential immunological or other factors influencing patients’ responses.

However, we included participants with similar characteristics in all treatment groups, and the efficacy of the three protocols revealed a real-life scenario.

## 5. Conclusions

A longer steroid scheme does not correlate with a better outcome, does not reduce the number of relapses, and does not prevent steroid failure.

The SiNePe protocol was associated with a lower incidence of FR- or SD-NS, but no statistically significant differences were found when this protocol was compared to the other two steroid treatments. In our cohort, no specific predictor factors of FR- or SD-NS were revealed. Furthermore, experiencing side effects during the first steroid treatment could be a good clinical marker of therapy response.

Further studies involving more centers are needed to evaluate different steroid protocols and patient outcomes.

## Figures and Tables

**Table 1:** Clinical characteristics of enrolled patients according to steroid regimen.

	All Patients	8 W	12 W	20 W
Patients, n	120	40	40	40
Steroid dose, mg/m^2^	-	2240 ± 2.9	3360 ± 2.9	3640 ± 2.9
Remission time, days	-	130	120	152
FR-NS, n (%)	59 (49)	20 (50)	18 (45)	22 (55)
2–6 years of age first relapse, n (%)	24 (73)	20 (100)	13 (72)	18 (82)
Number of relapses, mean ± SD	3.7 ± 2.6	3.2 ± 2.9	3.2 ± 2.3	4.2 ± 2.9
1–3 relapses, n (%)	19 (16)	10 (50)	10 (56)	9 (41)
>4 relapses, n (%)	31 (26)	10 (50)	8 (44)	13 (59)

Abbreviations: remission time: number of asymptomatic days after NS onset; FR-NS: relapsing nephrotic syndrome.

## Data Availability

The dataset generated and analyzed during the current study is available from the corresponding author on reasonable request.
